# High Prevalence of Antimicrobial-resistant Gram-negative Colonization in Hospitalized Cambodian Infants

**DOI:** 10.1097/INF.0000000000001187

**Published:** 2016-07-20

**Authors:** Paul Turner, Sreymom Pol, Sona Soeng, Poda Sar, Leakhena Neou, Phal Chea, Nicholas PJ Day, Ben S. Cooper, Claudia Turner

**Affiliations:** From the * Cambodia Oxford Medical Research Unit, Angkor Hospital for Children, Siem Reap, Cambodia; † Mahidol-Oxford Tropical Medicine Research Unit, Faculty of Tropical Medicine, Mahidol University, Bangkok, Thailand; ‡ Centre for Tropical Medicine and Global Health, Nuffield Department of Medicine, University of Oxford, Oxford, United Kingdom; and § Angkor Hospital for Children, Siem Reap, Cambodia.

**Keywords:** colonization, neonate, infant, Gram-negative, antimicrobial resistance

## Abstract

**Background:**

Antimicrobial-resistant Gram-negative infections are a significant cause of mortality in young infants. We aimed to determine characteristics of, and risk factors for, colonization and invasive infection caused by 3rd generation cephalosporin (3GC) or carbapenem-resistant organisms in outborn infants admitted to a neonatal unit (NU) in Cambodia.

**Methods:**

During the first year of operation, patients admitted to the Angkor Hospital for Children NU, Siem Reap, Cambodia, underwent rectal swabbing on admission and twice weekly until discharge. Swabs were taken also from 7 environmental sites. Swabs were cultured to identify 3GC or carbapenem-resistant *Acinetobacter* sp., *Escherichia coli*, *Klebsiella pneumoniae* and *Pseudomonas aeruginosa*.

**Results:**

The study included 333 infants with a median age at NU admission of 10 days (range, 0–43). Colonization by ≥1 3GC-resistant organism was detected in 85.9% (286/333). Admission swabs were collected in 289 infants: 61.9% were colonized by a 3GC-resistant organism at the time of admission, and a further 23.2% were colonized during hospitalization, at a median of 4 days [95% confidence interval: 3–5]. Probiotic treatment (hazard ratio: 0.58; 95% confidence interval: 0.35–0.98) was associated with delayed colonization. Colonization by a carbapenem-resistant organism occurred in 25 (7.5%) infants. Six infants had NU-associated *K. pneumoniae* bacteremia; phenotypically identical colonizing strains were found in 3 infants. Environmental colonization occurred early.

**Conclusions:**

Colonization by antimicrobial-resistant Gram-negative organisms occurred early in hospitalized Cambodian infants and was associated with subsequent invasive infection. Trials of potential interventions such as probiotics are needed.

## Abstract 2

Supplemental Digital Content is available in the text.

Infection caused by antimicrobial-resistant Gram-negative organisms has become a global health concern.^1^ In particular, *Klebsiella pneumoniae*, *Escherichia coli*, *Pseudomonas aeruginosa* and *Acinetobacter* species have been shown to be important causes of both community-acquired and hospital-acquired sepsis in neonates and infants in low- and middle-income countries.^2,3^ These organisms are frequently resistant to multiple antimicrobial classes.^4^ Lack of availability of second-line drugs, such as the carbapenems, significantly limits treatment options in resource poor setting and may result in increased mortality from these infections.^5,6^ Recent studies in Asia have highlighted the importance of these organisms in the region, most notably antimicrobial-resistant strains of *K. pneumoniae*.^7,8^ Previously published Cambodian pediatric bloodstream infection data from 2007 to 2011 demonstrated a high prevalence of 3rd generation cephalosporin (3GC) resistance in both community- and hospital-acquired *K. pneumoniae* infections.^6^ Despite the high burden of disease, little is known about the dynamics of colonization with these organisms amongst infants in low income settings. An understanding of such dynamics is important for the development of urgently needed strategies to contain these organisms.^1,9^

It is known that colonization of the gastrointestinal tract precedes invasive infection and provides a reservoir of organisms for transmission within the hospital setting.^10–13^ Risk factors for acquisition of these organisms in hospital have included prematurity, low birth weight, invasive devices (eg, feeding tubes), length of stay and antimicrobial exposure.^4,10,12,14,15^ The relative importance of early vertical transmission in neonatal colonization and infection is unknown.^16^ Importantly, most previous studies of colonization in neonates and infants have focused on neonatal intensive care units (NICUs) with high proportions of both inborn and premature infants. The prevalence of colonization by resistant Gram-negative organisms in neonates and infants admitted to hospital from the community is poorly defined. This is a significant knowledge gap, given that colonization represents a relatively hidden, but easily studied, reservoir of organisms.

The aim of the current study was to determine the prevalence and temporal characteristics of colonization by 3GC- or carbapenem-resistant *K. pneumoniae/oxytoca*, *E. coli*, *P. aeruginosa* and *Acinetobacter* species in outborn neonates and infants admitted to a new neonatal care unit in Cambodia, as a prelude to possible intervention studies.

## MATERIALS AND METHODS

### Study Design and Participants

The study was carried out at Angkor Hospital for Children (AHC), a nongovernmental paediatric hospital located in Siem Reap, Cambodia. Cambodia has neonatal and infant mortality rates of 17.6 and 33/1000 live births, respectively.^17^ The hospital provides secondary and tertiary level care to children 0–15 years of age and has no maternity unit. The neonatal unit (NU) comprises a high acuity area (NICU) and an adjacent lower acuity area (special care baby unit). All NU admissions were eligible for study enrolment during its first year of operation (September 11, 2013, to September 10, 2014). Patients could be reenrolled if they were readmitted to the NU after discharge to another ward. Data regarding the antenatal and delivery history; medical care before admission; and clinical, laboratory results, management and outcome at AHC were recorded in a case record form. Patients could be given an oral probiotic formulation (*Lactobacillus acidophilus*; Biorée granules, Daehan NewPharm, Seoul, South Korea) on admission to reduce the risk of necrotizing enterocolitis at the discretion of the treating clinician.

Written consent was obtained from mothers before study enrolment. The study was reviewed and approved by the AHC Institutional Review Board (1055/13 AHC) and the Oxford Tropical Ethics Committee (1047-13).

### Specimens and Laboratory Assays

Rectal swabs (Medical Wire and Equipment, Corsham, United Kingdom) were obtained from participants within 24 hours of admission and then twice weekly until NU discharge when a final swab was taken. Swabs were placed into Amies transport medium with charcoal and transported immediately to the hospital microbiology laboratory where they were inoculated onto MacConkey agar (Oxoid, Basingstoke, United Kingdom). The inoculum was streaked over the plate in 4 directions to create an even distribution of colonies. Ten microgram disks of cefpodoxime or imipenem (Oxoid) were added to the plates before overnight incubation in air at 37°C. Cefpodoxime or imipenem-resistant organisms were subcultured onto Columbia agar for phenotypic identification of target organisms by Gram stain, oxidase test (Remel, Lenexa, KS), biochemical tests [triple sugar iron agar, urea agar, citrate agar, motility-indole-lysine agar (Oxoid/BD, Franklin Lakes, NJ)] and API 20E/20NE (bioMerieux, Marcy L’Etoile, France), as appropriate. All non-*Acinetobacter baumannii* isolates were designated *Acinetobacter* sp. Antimicrobial susceptibilities were done by disk diffusion on Mueller-Hinton agar (Oxoid) using 2013 Clinical and Laboratory Standards Institute criteria.^18^ Drugs tested focused on those appropriate for parental treatment of infections in neonates and infants: ampicillin, ceftriaxone, ceftazidime, gentamicin and imipenem. Intermediate resistance was classified as susceptible in the following analyses, to give a conservative estimate of resistance as minimum inhibitory concentration data were not available. Extended spectrum β-lactamase (ESBL) production was determined for *E. coli* and *K. pneumoniae/oxytoca* isolates using the double-disk method [cefotaxime +/− clavulanate and ceftazidime +/− clavulanate (BD)], following Clinical and Laboratory Standards Institute guidelines. Carbapenemase activity was not formally tested in isolates displaying imipenem resistance. Quality control for culture media was done immediately after preparation of each new batch of plates, and antimicrobial discs were tested on a weekly basis using the appropriate American Type Culture Collection control strains.

Clinical culture specimens (ie, blood cultures) were taken at the discretion of the treating clinician. Bloodstream infections were defined as community-acquired if the blood was collected for culture ≤48 hours of AHC admission and hospital-acquired if collection was >48 hours after admission.

Environmental swabs were obtained from 7 sites on the NU at weekly intervals throughout the study period. The dry swab tip was rotated over the surface of the site before being placed into transport medium. Swab processing was identical to that described for rectal swabs.

### Data Analysis

Data were analyzed with the R statistical package version 3.2 (R Foundation for Statistical Computing, Vienna, Austria). Continuous data were compared using the Wilcoxon rank-sum test. Categorical data were compared using the χ^2^ or Fisher exact test. A multivariable logistic regression model was used to predict factors associated with colonization at first NU admission and included all variables from the univariable analysis. Time to colonization during the first NU admission was assessed using survival analysis, and association with timing of colonization was tested using univariable and multivariable Cox proportional hazards models. Antimicrobial drug exposures and feeding modalities were coded as time-varying covariates. Model fit was assessed using the Hosmer–Lemeshow test and examination of scaled Schoenfeld residuals, as appropriate.

### Role of the Funding Source

The sponsors of the study had no role in study design, data collection, data analysis, data interpretation or writing of the report. The corresponding author had full access to all the data in the study and had final responsibility for the decision to submit for publication.

## RESULTS

### Study Population

The study included 333 infants. The majority of infants were born at term (279; 83.8%), the median birth weight was 2.8 kg (range, 0.7–4.5), and 177 (43.2%) were male. All infants were outborn: 160 (48.0%) were born in hospital, 128 (38.4%) in a government health center, 21 (6.3%) at home and 24 (7.2%) in various other locations. The median age on first admission to the NU was 10 days (0–43), and 323 (97.0%) of first admissions were neonates (aged ≤28 days). Fifty-five infants (16.5%) had been admitted to another hospital before AHC, and 91 (27.3%) were admitted to another ward within AHC before NU admission. The median duration of the first NU admission was 5 days (0–65). Infants were classified as severe (ie, requiring ventilatory support or inotropes at any point of their AHC admission) in 67 (20.1%) cases. Nineteen (5.7%) infants were admitted to the NU more than once. Six (1.8%) infants died during admission, and treatment was withdrawn with the child taken home to die in 4 (1.2%) cases.

A total of 432 blood cultures were taken from 309 of the infants: 297 (68.8%) collected during the NU admission, 112 (25.9%) collected before NU admission and 23 (5.3%) collected after first NU discharge but before hospital discharge. Significant pathogen(s) were grown from 20 (5.3%) of cultures: 8 (40.0%) were considered community-acquired and 12 (60.0%) were hospital-acquired infections. *K. pneumoniae* was the commonest organism isolated [7/23 (30.4%) significant isolates], and all were 3GC-resistant and ESBL-positive (Table, Supplemental Digital Content 1, http://links.lww.com/INF/C462). All but 1 (6/7; 85.7%) of the *K. pneumoniae* bloodstream infections were hospital-acquired.

### Infant Colonization

A median of 4 (1–22) rectal swabs were collected per infant. Overall, 286 (85.9%) infants were colonized by at least 1 3GC-resistant organism [most commonly *K. pneumoniae/oxytoca* (253; 76.0%)], and 25 (7.5%) were colonized by an imipenem-resistant organism [most commonly *A. baumannii* (19; 6.0%)]. Over half (161/286; 56.3%) were colonized by more than 1 3GC-resistant species. *P. aeruginosa* colonization was rarely detected, with only 1 infant colonized at a single time point. ESBLs were detected in almost all 3GC-resistant *E. coli* (555/573; 96.9%) and *K. pneumoniae/oxytoca* (1412/1433; 98.5%). Gentamicin resistance was also common in these organisms, being detected in 89.7% (35/39) of *A. baumannii*, 58.2% (334/574) of *E. coli* and 57.9% (829/1433) of *K. pneumoniae/oxytoca* isolates.

An admission swab was obtained from 289 infants, and a 3GC-resistant target organism was cultured from 179 (61.9%) of these swabs. Colonization at admission did not vary by pre-NU admission status: 62.6% (102/163) in those admitted direct to the NU compared with 60.3% (47/78) in those initially admitted to another AHC department or 62.5% (30/48) in those transferred from another hospital. Of the infants noncolonized on admission, 67 (23.2% of all 289 infants with an admission swab; 60.9% of the 110 infants noncolonized on admission) were subsequently colonized by at least one of the target organisms during the first admission to the NU (Table 1 and Fig., Supplemental Digital Content 2, http://links.lww.com/INF/C463). Of the infants who did not have an admission swab, 38/44 (86.4%) were colonized but the timing of first colonization could not be confirmed. In a multivariable logistic regression model, delivery in hospital was associated with colonization on first admission to the NU [odds ratio: 3.03, 95% confidence interval (CI): 1.73–5.37], and there was a similar trend for those infants born after prolonged rupture of membranes (3.79, 0.99–24.97) or having a more severe illness (2.10, 0.96–4.78; Table 2).

**TABLE 1.**
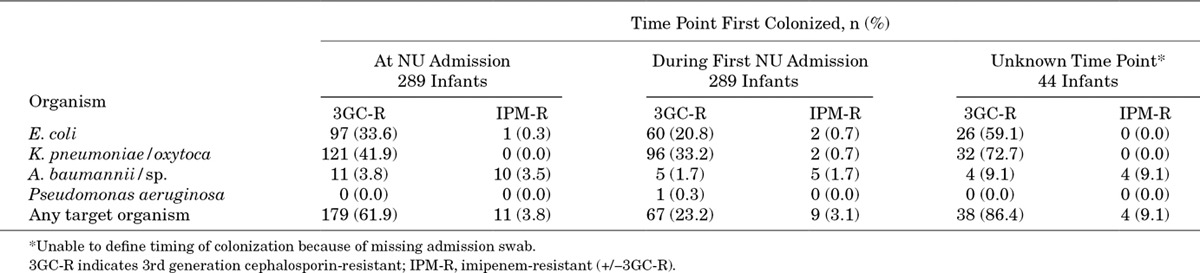
Colonization by Antimicrobial-resistant *Acinetobacter baumannii*/sp., *Escherichia coli* and *Klebsiella pneumoniae/oxytoca* in 333 Hospitalised Cambodian Young Infants

**TABLE 2.**
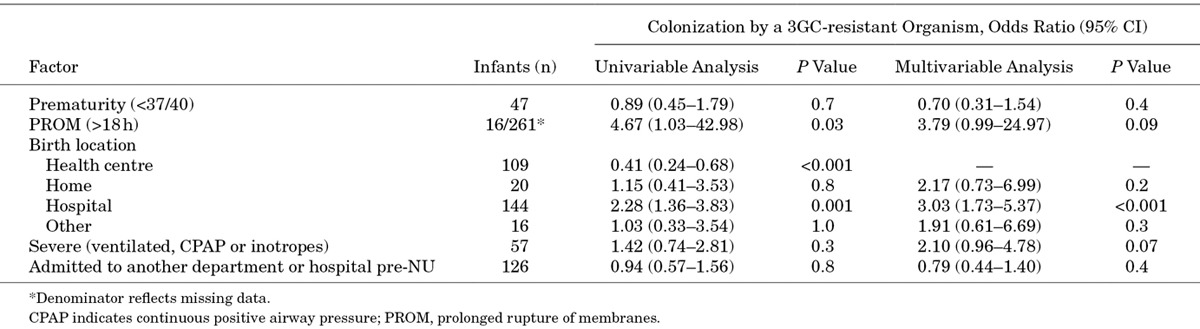
Results of Univariable and Multivariable Logistic Regression Models Exploring Factors Associated With Colonization by 3rd Generation Cephalosporin-resistant *Acinetobacter baumannii*/sp., *Escherichia coli, Klebsiella pneumoniae/oxytoca* or *Pseudomonas aeruginosa* on First Admission to the NU for 289 Infants

In the 110 infants known to be colonization free on NU admission, the median time to colonization by a 3GC-resistant organism in 67 infants was 4 days (95% CI: 3–5; Fig. 1). In univariable Cox proportional hazards models, breast milk exposure [hazard ratio (HR): 0.39, 95% CI: 0.18–0.84] and probiotic treatment (0.57, 0.35–0.93) were associated with delayed colonization by a 3GC-resistant organism during the first NU admission (Fig. 2; Table 3). Formula milk exposure was associated with earlier colonization (1.68, 1.03–2.74; Table 3). In the multivariable model, only being probiotic treated (0.58, 0.35–0.98) remained associated with delayed colonization. Antimicrobial drug exposure, mostly gentamicin (coadministered with ampicillin in 63/66 infants), did not impact timing of colonization (Table 3).

**TABLE 3.**
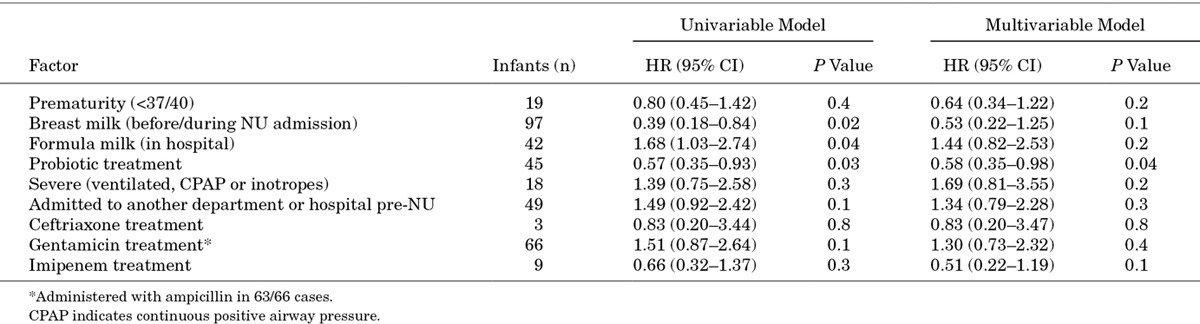
Results of Univariable and Multivariable Cox Proportional Hazards Models to Define Factors Affecting Time to Colonization by a 3rd Generation Cephalosporin-resistant *Acinetobacter baumannii*/sp., *Escherichia coli, Klebsiella pneumoniae/oxytoca* or *Pseudomonas aeruginosa* Isolate in 110 Infants Admitted to the NU and Found to be Noncolonized on Admission

**FIGURE 1.**
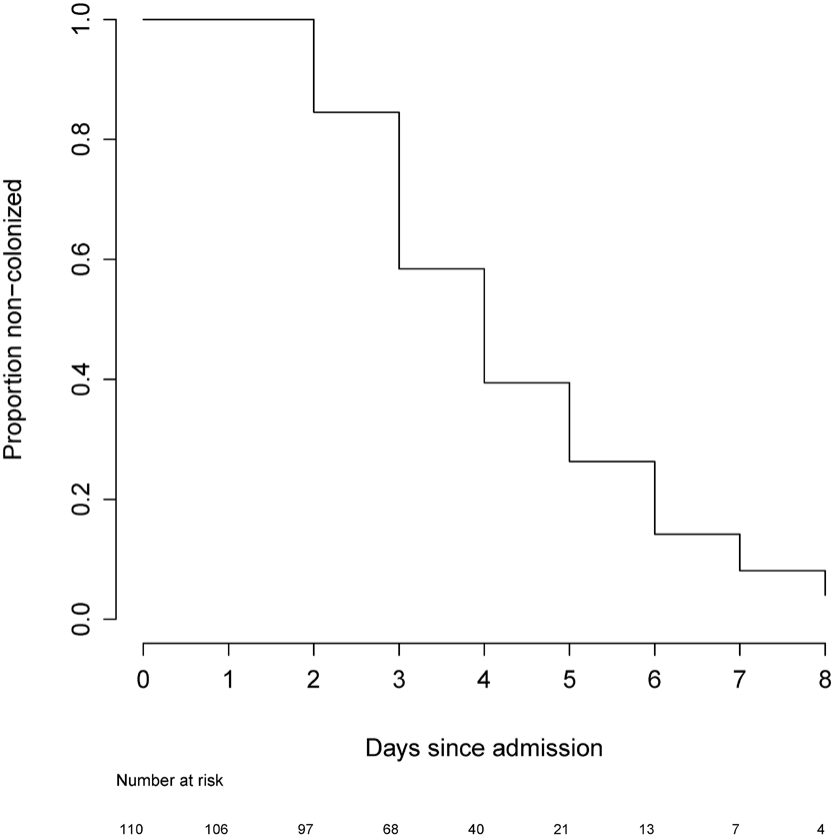
Time to colonization by a 3rd generation cephalosporin-resistant organism in 110 infants admitted to the NU and found to be noncolonized on admission

**FIGURE 2.**
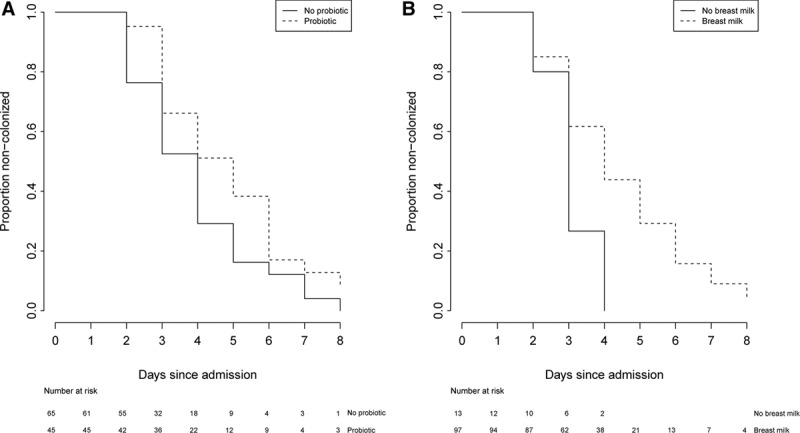
The effect of oral probiotic exposure (A) or breast milk consumption (B) on colonization by a 3rd generation cephalosporin-resistant organism in 110 infants admitted to the NU and found to be noncolonized on admission. Forty-five of the infants were given a *L. acidophilus*-containing oral probiotic formulation as part of their clinical management. Ninety-seven infants received breast milk.

Rectal swabs were taken before blood culture in 4/6 of the infants who had *K. pneumoniae* bacteremia during their NU admission: colonizing *K. pneumoniae* with identical antibiograms to the blood culture isolate were found in 3 of the infants.

### Environmental Colonization

A total of 362 environmental swabs were obtained from 7 sampling locations. One or more of the target organisms were isolated from 148 (40.9%) of these swabs. The proportion of positive swabs varied by site, from 4.0% (2/50) for the NICU computer keyboard to 96.2% (50/52) for the milk kitchen sink (Table, Supplemental Digital Content 3, http://links.lww.com/INF/C464). Environmental colonization occurred early. All sites were colonized by 3GC-resistant organisms within 3 months of the unit opening, and 3 (42.9%; dirty utility sink, milk kitchen sink, and isolation room sink) were colonized by an imipenem-resistant organism (Table, Supplemental Digital Content 4, http://links.lww.com/INF/C465). Patterns of colonization varied, with the dirty utility and milk kitchen sinks being persistently colonized but the NICU computer keyboard being rarely so (Fig., Supplemental Digital Content 5, http://links.lww.com/INF/C466).

## DISCUSSION

This study has demonstrated the high prevalence of antimicrobial-resistant Gram-negative colonization in outborn but hospitalized Cambodian neonates and infants. The new NU environment was rapidly colonized by the same organisms. In 3/4 cases where it could be assessed, we observed that infants with *K. pneumoniae* bacteremia were colonized by phenotypically identical strains before infection onset. Colonization occurred before admission to the NU in almost two-thirds of infants. Being born in a hospital was associated with a greatly increased risk of early colonization. An association between prolonged rupture of membranes and early colonization suggests that vertical transmission of resistant Gram-negative organisms may be responsible for a proportion of early infant colonization, although supporting data for this hypothesis are limited.^16^ Treatment with a *L. acidophilus*-based probiotic reduced the rate of colonization by over one-third.

While the entirely outborn patient population makes comparison with data from other NUs difficult, the factors associated with colonization during NU stay are in broad agreement with data from studies on NUs with patient populations dominated by inborn premature infants.^4,13,15^ However, the overall prevalence of colonization admission was far higher than that reported in many previous studies: 86% infants were colonized by at least 1 3GC-resistant organism. For example, only 1% of transferred neonates were colonized by antimicrobial-resistant Gram-negative organisms on admission to a US NU,^19^ and 11%–24% of Israeli neonates acquired ESBL-positive *K. pneumoniae* colonization during NU admission.^20^ An Indian study found that 97/238 (41%) infants were colonized by ESBL-producing *E. coli* during NU admission.^14^ In contrast to our findings, a previous nonoutbreak-related study of environmental colonization of a new NU in the United States found a predominance of skin (eg, coagulase-negative *staphylococci*) and water-associated organisms (eg, *P. aeruginosa*); coliforms and *Acinetobacter* sp. were not identified.^21^

The potential impact of probiotics on colonization is currently not well defined. There is increasing evidence that probiotic use in premature neonates reduces the risk of developing necrotizing enterocolitis.^22^ However, a previous small study noted no effect of *L. acidophilus* on gastrointestinal colonization of premature infants,^23^ and a recent study on infants with colic showed no impact of *Lactobacillus reuteri* on gastrointestinal microbiome diversity.^24^ However, on-going large neonatal studies, including the UK-based PiPS trial of *Bifidobacterium breve* BBG, will include analyses of impact of probiotics on bloodstream infections and gastrointestinal colonization by resistant organisms. Recent UK guidelines for management and prevention of Gram-negative infection outbreaks in NUs highlighted the need for research into areas such as the utility of routine surveillance swabs for identification of colonization/resistance and to determine whether probiotics may affect Gram-negative bacteremia incidence.^25^ Given the significant variation in patient populations and colonization/antimicrobial resistance prevalence, the findings of all such studies would need to be validated in appropriate low-income settings including those admitting predominantly outborn infants.

There were several limitations to our study. A rectal swab was not obtained from the mothers of participants, and thus it was not possible to formally assess the proportion of colonization that was attributable to vertical transmission. The laboratory methodology used, although similar to other studies,^10,12,26^ may have underestimated the prevalence of colonization. In future studies, the use of chromogenic ESBL or carbapenemase screening media would be preferable. To further study the links between environmental and patient colonization and development of invasive infection, molecular characterization of the isolates would have been desirable and such follow-up work is planned. Finally, the study was conducted in a single site, and thus extrapolation of the results to the wider region must be done cautiously. However, the data give important insights into early gastrointestinal colonization by antimicrobial-resistant and disease-associated organisms in neonates and infants from a resource-poor setting with high neonatal and infant mortality.

In conclusion, gastrointestinal colonization by drug-resistant Gram-negative organisms occurred early in hospitalized Cambodian neonates and infants and was associated with subsequent invasive infection. We found probiotic use to be associated with a large reduction in the rate of acquisition of these organisms. This association is biologically plausible. Probiotics have not previously been assessed as a potential intervention for reducing acquisition of highly antimicrobial-resistant Gram-negative organisms in settings where such organisms are hyperendemic. In light of our findings, we believe assessment of this intervention with randomized trials should be given high priority in such settings.

## Supplementary Material
