# Supplementary material for: High Prevalence of Antimicrobial-resistant Gram-negative Colonization in Hospitalized Cambodian Infants
Source: Pediatr Infect Dis J. 2016 Jul 20;35(8):856–61. doi: 10.1097/INF.0000000000001187 (PMC4957964; doi:10.1097/INF.0000000000001187)
Supplement: Supplementary file 1 [file inf-35-856-s001.docx]

**Table 1. Colonization by antimicrobial-resistant *Acinetobacter baumannii/*sp.*, Escherichia coli, and Klebsiella pneumoniae/oxytoca* in 333 hospitalised Cambodian young infants**

| **Organism** | **Time point first colonized, n (%)** | | | | | |
| --- | --- | --- | --- | --- | --- | --- |
|  | **At NU* admission** | | **During NU stay(s)** | | **Unknown time point**† | |
|  | **289 infants** | | | | **44 infants** | |
|  | **3GC-R**ǂ | **IPM-R**ǂ | **3GC-R** | **IPM-R** | **3GC-R** | **IPM-R** |
| *E. coli* | 97 (33.6) | 1 (0.3) | 60 (20.8) | 2 (0.7) | 26 (59.1) | 0 (0.0) |
| *K. pneumoniae/oxytoca* | 121 (41.9) | 0 (0.0) | 96 (33.2) | 2 (0.7) | 32 (72.7) | 0 (0.0) |
| *A. baumannii/*sp. | 11 (3.8) | 10 (3.5) | 5 (1.7) | 5 (1.7) | 4 (9.1) | 4 (9.1) |
| *P. aeruginosa* | 0 (0.0) | 0 (0.0) | 1 (0.3) | 0 (0.0) | 0 (0.0) | 0 (0.0) |
| Any target organism | 179 (61.9) | 11 (3.8) | 67 (23.2) | 9 (3.1) | 38 (86.4) | 4 (9.1) |

*NU: neonatal unit

†Unable to define timing of colonization due to missing admission swab

ǂ3GC-R: 3^rd^ generation cephalosporin-resistant; IPM-R: imipenem-resistant (+/- 3^rd^ generation cephalosporin-resistant)
