# Supplementary material for: High Prevalence of Antimicrobial-resistant Gram-negative Colonization in Hospitalized Cambodian Infants
Source: Pediatr Infect Dis J. 2016 Jul 20;35(8):856–61. doi: 10.1097/INF.0000000000001187 (PMC4957964; doi:10.1097/INF.0000000000001187)
Supplement: Supplementary file 2 [file inf-35-856-s002.docx]

**Table 2. Results of univariable and multivariable logistic regression models exploring factors associated with colonization by 3^rd^ generation cephalosporin-resistant *A. baumannii/*sp., *E. coli, K. pneumoniae/oxytoca,* or *P. aeruginosa* on first admission to the neonatal unit for 289 infants**

| **Factor** | **Infants (n)** | **Colonization by a 3GC*-resistant organism, OR (95% CI)** | | | |
| --- | --- | --- | --- | --- | --- |
|  |  | **Univariable analysis** | **P-value** | **Multivariable analysis** | **P-value** |
| Prematurity (<37/40) | 47 | 0.89 (0.45 – 1.79) | 0.7 | 0.70 (0.31 – 1.54) | 0.4 |
| PROM (>18 hours)† | 16/261‡ | 4.67 (1.03 – 42.98) | 0.03 | 3.79 (0.99 – 24.97) | 0.09 |
| Birth location |  |  |  |  |  |
| *Health Centre* | 109 | 0.41 (0.24 – 0.68) | <0.001 | - | - |
| *Home* | 20 | 1.15 (0.41 – 3.53) | 0.8 | 2.17 (0.73 – 6.99) | 0.2 |
| *Hospital* | 144 | 2.28 (1.36 – 3.83) | 0.001 | 3.03 (1.73 – 5.37) | <0.001 |
| *Other* | 16 | 1.03 (0.33 – 3.54) | 1.0 | 1.91 (0.61 – 6.69) | 0.3 |
| Severe (ventilated, CPAP**, or inotropes) | 57 | 1.42 (0.74 – 2.81) | 0.3 | 2.10 (0.96 – 4.78) | 0.07 |
| Admitted to another department or hospital pre-NU†† | 126 | 0.94 (0.57 – 1.56) | 0.8 | 0.79 (0.44 – 1.40) | 0.4 |

* 3^rd^ generation cephalosporin-resistant

† PROM: prolonged rupture of membranes

‡ Denominator reflects missing data

** CPAP: continuous positive airway pressure

†† NU: neonatal unit
