# Supplementary material for: High Prevalence of Antimicrobial-resistant Gram-negative Colonization in Hospitalized Cambodian Infants
Source: Pediatr Infect Dis J. 2016 Jul 20;35(8):856–61. doi: 10.1097/INF.0000000000001187 (PMC4957964; doi:10.1097/INF.0000000000001187)
Supplement: Supplementary file 3 [file inf-35-856-s003.docx]

**Table 3. Results of univariable and multivariable Cox proportional hazards models to define factors affecting time to colonization by a 3^rd^ generation cephalosporin-resistant *A. baumannii/*sp., *E. coli, K. pneumoniae/oxytoca,* or *P. aeruginosa* isolate in 110 infants admitted to the neonatal unit and found to be non-colonized on admission**

| **Factor** | **Infants (n)** | **Univariable model** | | **Multivariable model** | |
| --- | --- | --- | --- | --- | --- |
|  |  | **HR (95% CI)** | **P-value** | **HR (95% CI)** | **P-value** |
| Prematurity (<37/40) | 19 | 0.80 (0.45 – 1.42) | 0.4 | 0.64 (0.34 – 1.22) | 0.2 |
| Breast milk (before / during NU* admission) | 97 | 0.39 (0.18 – 0.84) | 0.02 | 0.53 (0.22 – 1.25) | 0.1 |
| Formula milk (in hospital) | 42 | 1.68 (1.03 – 2.74) | 0.04 | 1.44 (0.82 – 2.53) | 0.2 |
| Probiotic treatment | 45 | 0.57 (0.35 – 0.93) | 0.03 | 0.58 (0.35 – 0.98) | 0.04 |
| Severe (ventilated, CPAP†, or inotropes) | 18 | 1.39 (0.75 – 2.58) | 0.3 | 1.69 (0.81 – 3.55) | 0.2 |
| Admitted to another department or hospital pre-NU | 49 | 1.49 (0.92 – 2.42) | 0.1 | 1.34 (0.79 – 2.28) | 0.3 |
| Ceftriaxone treatment | 3 | 0.83 (0.20 – 3.44) | 0.8 | 0.83 (0.20 – 3.47) | 0.8 |
| Gentamicin treatment‡ | 66 | 1.51 (0.87 – 2.64) | 0.1 | 1.30 (0.73 – 2.32) | 0.4 |
| Imipenem treatment | 9 | 0.66 (0.32 – 1.37) | 0.3 | 0.51 (0.22 – 1.19) | 0.1 |

*NU: neonatal unit

† CPAP: continuous positive airway pressure

‡Administered with ampicillin in 63/66 cases
